# Polyethylene-grafted poly(hexamethylene guanidine) modified ultra-high molecular weight polyethylene monofilaments and their antimicrobial properties

**DOI:** 10.1098/rsos.250340

**Published:** 2025-10-29

**Authors:** Wenyang Zhang, Hongzhan Song, Yongli Liu, Jin Chen, Jiangao Shi, Lingzhi Li

**Affiliations:** ^1^ East China Sea Fisheries Research Institute, Chinese Academy of Fishery Sciences, Shanghai, People’s Republic of China

**Keywords:** ultra-high molecular weight polyethylene monofilaments, polyethylene-grafted poly(hexamethylene guanidine), knot strength, antibacterial properties

## Abstract

Biofouling on netting poses a significant challenge, as it can considerably increase the weight of the nets and shorten their service lifespan. This study investigates the properties of polyethylene-grafted poly(hexamethylene guanidine) (PE-g-PHMG) modified ultra-high molecular weight polyethylene (UHMWPE) monofilaments. The results show that the addition of PE-g-PHMG decreases the degree of crystallinity, average lattice spacing and crystallite size of the blend monofilaments, leading to relatively low rigidity and high toughness. The incorporation of PE-g-PHMG enhances both the knot strength (increased by 17.9%) and the overall antibacterial performance (efficacy rates of 99.4% and 97.2% against *Escherichia coli* and *Staphylococcus aureus*) of the blend monofilaments. Furthermore, blend monofilaments (UHMWPE-20%) after washing 30 times exhibit high efficacy rates against *E. coli* and *S. aureus*. This article provides a method for preparing fishing grade polyethylene monofilaments that effectively combines favourable characteristics, including enhanced knot strength and good antibacterial properties.

## 1. Introduction

Net cages constitute a fundamental component of marine aquaculture infrastructure. High-density polyethylene (HDPE) knotted netting is the material of choice for the construction of these net cages, owing to its advantageous performance-to-cost ratio, ease of processing and exceptional resistance to moisture and degradation over time [1]. The ability of these nets to withstand the combined forces of wind, waves and currents in marine environments is intrinsically linked to their safety performance. However, extended exposure to seawater results in considerable biofouling, as a variety of marine organisms adhere to the nets. This accumulation significantly increases the initial weight of the nets, leading to their operation beyond the intended capacity and markedly diminishing their service life [2]. Additionally, the activities of fouling organisms can have erosive and destructive impacts on the nets themselves [3]. As a result, net biofouling has emerged as a significant concern in the maintenance and longevity of net cages in marine aquaculture.

In recent years, various antifouling strategies have been used to mitigate pollution associated with fishing nets [4–6]. Compared with traditional manual and mechanical cleaning methods [7,8], antifouling coating is currently the most widely used antifouling method. For instance, Ashraf & Edwin [9] reported that the nylon fishing cage net covered with antifouling coating, prepared by *in situ* synthesis of nano copper oxide incorporated inpoly(ethylene glycol) methacrylate based hydrogel, exhibits excellent fouling resistance with the lowest biomass accumulation after 90 days of exposure in the estuarine environment. The most commonly used antifoulant is Cu-based coatings, which are used in the maintenance of fish cages and nets [10]. However, the release of these Cu-based coatings and their potential for bioaccumulation have raised increasing concerns regarding their impact on benthic fauna and surrounding environment.

Polyhexamethylene guanidine hydrochloride (PHMG) has attracted considerable attention due to its effective broad-spectrum antibacterial properties, ease of graft modification and high thermal and chemical stability [11–16]. The antifouling mechanism of biocides within the guanidine family is primarily attributed to their cationic nature, which disrupts bacterial cell membranes through ionic interactions, resulting in membrane destruction, leakage of intracellular contents and subsequent bacterial death [17–19]. The high solubility of PHMG in water may facilitate its rapid dispersion in seawater. To enhance long-term antimicrobial effect, PHMG is chemically grafted onto polyethylene (PE) to prepare composite fibres [20] and fishing net materials with long-lasting antifouling properties. In previous research work, our research team has demonstrated that fishing nets coated with poly(hexamethylene guanidine)-modified polypropylene exhibit sustained antifouling characteristics over extended periods [21].

The mechanical properties of HDPE knotted netting are fundamentally associated with the strength of polyethylene monofilaments. As net cages are increasingly deployed in deeper marine environments, there is a growing demand for enhanced strength in polyethylene monofilaments, particularly with respect to knot strength. Knot strength is a critical parameter for evaluating the performance of fibres, especially in specialized applications such as fishing nets [22,23]. The integration of ultra-high molecular weight polyethylene (UHMWPE) with HDPE is expected to improve the tensile strength of monofilaments. Research conducted by Wang *et al.* [24] indicates that the blending of UHMWPE with HDPE can enhance both the tensile strength and initial modulus of the resulting fibres. However, the exploration of knot strength in UHMWPE monofilaments remains insufficiently addressed.

In the present study, we examine the impact of polyethylene-grafted poly(hexamethylene guanidine) (PE-g-PHMG) on the structural and functional characteristics of UHMWPE/PE-g-PHMG monofilaments. It has been established that both the knot strength and antibacterial efficacy of the blended monofilaments improve with increasing concentrations of PE-g-PHMG. Additionally, monofilaments demonstrating a combination of high knot strength and advantageous antibacterial properties were successfully developed. The interrelationship among the chemical structure, crystalline structure and the thermal, mechanical and antibacterial properties was comprehensively investigated.

## 2. Experimental section

### 2.1. Materials of modified ultra-high molecular weight polyethylene granules

The PE-g-PHMG was obtained from Guilin Prenovo Antibacterial Material Co., Ltd. (Guilin, China). This material is characterized as an environmentally friendly and broad-spectrum antibacterial material with a PHMG grafting rate of 20% and a melt flow index of 20 (190℃, g (10 min)^−1^). UHMWPE (LL-1040) with a *M_η_* of approximately 1.5 × 10^6^ was sourced from Shanghai Lianle Chemical Science and Technology Co., Ltd. (China). HDPE (5000 s) with an Melt Flow Index (MFI) of 0.9 g (10 min)^−1^ (190℃, 2.16 kg) was procured from Sinopec Yangzi Petrochemical Co., Ltd. (Nanjing, China). Thermal degradation, crystallinity and melting behaviour of the pristine polymers (HDPE, UHMWPE and PE-g-PHMG) are shown in electronic supplementary material, figures S1 and S2 and tables S1 and S2.

### 2.2. Preparation of modified ultra-high molecular weight polyethylene granules

Initially, a composite consisting of HDPE particles, UHMWPE powder (20 wt%), polyethylene wax (1 wt%) and antioxidants, Irganox 1010 and Irgafos 168 (0.2 wt% of each) was prepared through physical mixing in a high-speed mixer. This mixture was subsequently subjected to melt mixing at a temperature of 240℃ using a co-rotating twin-screw extruder (D = 40 mm, L/D = 44). Following this process, the continuous strands were converted into smaller granules using a dry strand pelletizer. The modified granules were dried overnight at 80℃ in an oven prior to melt spinning.

### 2.3. Preparation of blend monofilaments

Modified UHMWPE granules were melt-extruded with PE-g-PHMG (0, 10 and 20%) by a single-screw extruder after premixing sufficiently. The extrusion temperatures were set at 200, 228, 240, 245 and 245℃ from the feed zone to the output end of the extruder, and the spinneret temperature was set at 245℃. Besides, the length/diameter (L/D) ratio of single screw was 32 : 1, and the diameter of spinneret aperture was 1.0 mm. After the as-spun fibres were extruded, they were kept in cold water at 25℃, subsequently in a hot water tank at 98℃ and then stretched to different draw ratios. Finally, these blend monofilaments (named as UHMWPE, UHMWPE-10% and UHMWPE-20%) with different diameters were wound into a coil on the winding machine.

### 2.4. Characterization

Tensile tests were performed using a uniaxial testing system (Instron 4466, Instron Corp., MA, USA) that was equipped with a 500 N load cell and operated at a crosshead speed of 250 mm min^−1^. A minimum of seven samples for each composition were tested, and the average values of the mechanical parameters were calculated from the tensile curves.

The melting and crystallization behaviour of monofilaments was examined using a differential scanning calorimeter (DSC 204F1 Phoenix, Netzsch Instruments, Germany). The heating and cooling rates were consistently maintained at 10℃ min^−1^, with the temperature range set from 30 to 240℃. The degree of crystallinity was calculated using the following equation:


$$X_{c}=\frac{\Delta H}{\Delta H_{m}^{0}},$$

where *ΔH* is the melting enthalpy of investigated monofilaments and is the melting enthalpy of 100% crystalline PE taken as 293 J g^−1^ [25].

Infrared (IR) spectroscopy in the attenuated total reflection (ATR) mode was carried out to investigate the structure of monofilaments using Fourier transform infrared spectroscope (FTIR) PerkinElmer Spectrum Two within the wave region of 4000–500 cm^−1^ and a resolution of 4 cm^−1^. Following the scanning process, the data were converted to relative absorbance values (A) according to the equation of log(1/T) and semi-quantitative infrared spectrum analysis was conducted using PerkinElmer Spectrum IR software. The intensity ratio of two absorption peaks, C=N stretching vibration peak (*I*_1640_) and C-H bending vibration peak (*I*_1467_), was derived from the analysis and used to quantify the PHMG content.

The X-ray diffraction (XRD) measurements were performed at room temperature using Cu Kα-irradiation (D8 advance, Bulker, Germany). The measurement range for 2*θ* was set between 5° and 35°, with a scanning speed of 3° min^−1^. The XRD profiles were analysed and fitted to multiple crystalline peaks, in addition to one amorphous halo. The average lattice spacing was determined in accordance with Bragg’s equation [26],


$$d_{hkl}=\frac{\lambda }{2\sin\theta },$$

and the average crystallite size was estimated by using the Scherrer equation [27,28],


$$D_{hkl}=\frac{K\lambda }{(\Delta 2\theta )\times \cos\theta },$$

where *d*_*hkl*_ represents the interplanar spacing between different diffraction planes, *D*_*hkl*_ indicates the crystallite size measured along the normal direction of the (*hkl*) plane diffraction. The symbol (*λ*) denotes the wavelength, (Δ2*θ*) refers to the full width at half maximum (FWHM) of the diffraction peak (*hkl*), measured in radians. The shape factor (*K*) is assigned a value of 0.9 for polymer systems and *θ* is defined as half of the diffraction angle.

The dynamic mechanical analyser (DMA, model 242C, Netzsch Instruments, Selb, Germany) was used to examine the viscoelastic properties of monofilaments. The measurements were performed in tensile mode, with the temperature ranging from −50 to 120℃, at a constant frequency of 1 Hz and a heating rate of 3℃ min^−1^.

Thermogravimetric analysis (TGA) was performed using a thermogravimetric analyser (TGA4000, PerkinElmer Co., Ltd., USA) to evaluate the thermal stability of the samples. Each sample, weighing between 7 and 8 mg, was subjected to a heating protocol that increased the temperature from 30℃ to 600℃ at a rate of 20℃ min^−1^, under a nitrogen flow of 50 ml min^−1^.

The shaking flask method was used to evaluate the antimicrobial properties of monofilaments. Gram-negative bacteria (*Escherichia coli,* ATCC 25922) and Gram-positive bacteria (*Staphylococcus aureus,* ATCC 6538) were cultured in an incubator at 37℃ for a duration of 24 h. Following this bacterial activation phase, the blend monofilaments (0.5 g) were sectioned into segments and introduced into a centrifuge tube containing 5 ml of diluted bacterial suspension and 100 μl of the diluted bacterial solution, respectively. Subsequently, the centrifuge tubes were incubated on a shaking platform at 120 r.p.m. and 37℃ for 18 h. After the incubation period, 100 µl of the diluted bacterial suspension (concentration of approx. 10^5^ CFU ml^−1^) was plated onto Luria-Bertani (LB agar plates, which were then placed in a bacterial incubator at 37℃. The number of colonies that developed was quantified, and the inhibition ratios were calculated using the following equation [29]:


inhibition ratio(%)=(C−E)/C×100,

where *E* is the colony number formed in the UHMWPE monofilament and *C* is the colony number formed in the blend monofilament. To evaluate the retention of antimicrobial properties after the post-wash durability, the blend monofilaments were tested for laundering durability of antibacterial effect after 30 cycles washing. Blend monofilaments were laundered with the machine set for warm water (40℃) at normal cycle. After laundering, they were rinsed with distilled water and then dried at room temperature.

## 3. Results and discussion

### 3.1. The drawability of as-spun monofilaments

The drawability, defined as the capacity to elongate fibres without fracture, is a critical parameter that characterizes the drawing process of as-spun monofilaments and is generally evaluated by the maximum achievable draw ratio [30]. To achieve high tensile strength, a high draw ratio is essential due to its critical influence on the condensed structure of blend monofilaments. As shown in table 1, the maximum achievable draw ratio of as-spun blend monofilaments varies considerably. A maximum draw ratio of 10 could be attained with UHMWPE monofilaments, whereas significant reduction in the maximum draw ratio (DR = 8) for UHMWPE-10% and UHMWPE-20% can be seen in table 1, suggesting the addition of PE-g-PHMG has significant effects on drawability.

**Table. 1.** The drawability of as-spun monofilaments.

samples	draw ratio (DR)		
	8	9	10
UHMWPE	spinning smoothly	spinning smoothly	spinning smoothly
UHMWPE-10%	spinning smoothly	breakage	breakage
UHMWPE-20%	spinning smoothly	breakage	breakage

### 3.2. Tensile behaviour

**Figure 1.**
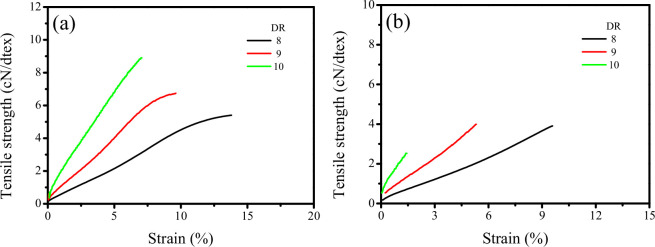
Tensile curves of knotless (a) and knotted (b) UHMWPE monofilaments with different draw ratios.

The breaking strength and strain of monofilaments serve as critical performance indicators for fishing net materials, significantly influencing both the service life and safety of fishing nets. The tensile curves of knotless and knotted UHMWPE monofilaments with varying draw ratios are depicted in figure 1. Specifically, figure 1a demonstrates that the knotless UHMWPE monofilament with a draw ratio of 8 exhibiting an S-shaped curve, characterized by a gradual increase in tensile strength up to approximately 5% strain, followed by a rapid escalation in tensile strength. In contrast, knotless UHMWPE monofilaments with increasing draw ratios consistently exhibit a positive correlation with tensile strength. Conversely, the knotted monofilaments, regardless of draw ratio, display only a marginal increase in tensile strength as strain.

**Figure 2.**
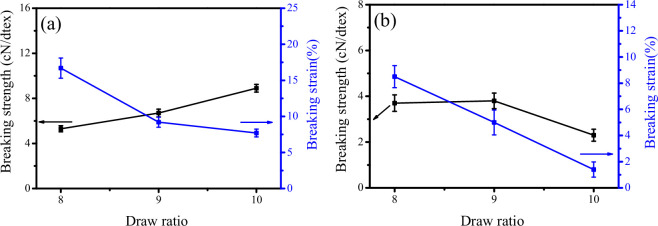
Breaking strength and strain of knotless (a) and knotted (b) UHMWPE monofilaments at different draw ratios.

It is well established that the breaking strength of knotless monofilaments increases with an increasing draw ratio, while the breaking strain exhibits markedly different trends, as illustrated in figure 2a. The breaking strain serves as a critical mechanical parameter indicative of the ductility of monofilaments. As the draw ratio increases, the ductility of the monofilament progressively diminishes. The knot strength and strain of UHMWPE monofilaments at various draw ratios are presented in figure 2b. Notably, the knot strain demonstrates a significant, approximately linear decrease with increasing draw ratio. It is important to highlight that knot strength does not exhibit a consistent increase with rising draw ratios; rather, it initially experiences a slight increase before sharply declining at higher draw ratios (specifically at DR = 10). This phenomenon can be attributed to the high draw ratio inducing greater orientation of polymer chains, which in turn enhances crystallinity, as evidenced in electronic supplementary material, figures S3, while also increasing the rigidity of the monofilament. However, this increased rigidity comes at the expense of toughness to a certain degree. The insufficient toughness of the monofilament at elevated draw ratios results in the stress concentration area near the knot being unable to undergo effective plastic deformation, which is inadequate for supporting the transmission of force, ultimately leading to fracture.

Based on the mechanical responses of both knotless and knotted UHMWPE monofilaments at various draw ratios, a draw ratio of 8 was selected as a reference point for further investigation into the effects of PE-g-PHMG content on the structure and properties of blended monofilaments.

### 3.3. Fourier transform infrared analysis

**Figure 3.**
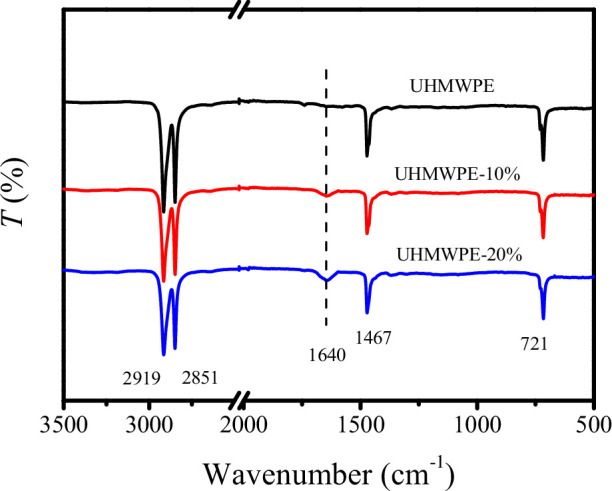
FTIR spectra of UHMWPE/PE-g-PHMG monofilaments in the wavenumber range of 3500−500 cm^−1^.

To elucidate the influence of PE-g-PHMG content on the chemical structure, FTIR spectra of UHMWPE/PE-g-PHMG monofilaments within the wavenumber range of 3500−500 cm^−1^ are presented in figure 3. The spectra reveal C-H stretching vibration peaks at 2919 and 2851 cm^−1^, a C-H bending vibration peak at 1467 cm^−1^ and a C-H rocking vibration peak at 721 cm^−1^ in the UHMWPE monofilament. In contrast to the UHMWPE monofilament, a new absorption peak at 1640 cm^−1^, attributed to the carbon nitrogen double bond (C=N) of PHMG, is evident in the blend monofilaments. This observation indicates that PHMG was successfully grafted onto PE and remains present in the blend monofilaments following high-temperature melt spinning. To quantify the PHMG content in the blend monofilaments, the intensity ratios (*I*_1640_/*I*_1467_) were used for estimation. The FTIR absorbance spectra are shown in electronic supplementary material, figure S4. The intensity ratio (*I*_1640_/ *I*_1467_) of UHMWPE-10% monofilament is 15.7%, while the intensity ratio of UHMWPE-20% monofilament is 23.2%, suggesting that the relative content of antibacterial components increases with increasing PE-g-PHMG content.

### 3.4. Thermogravimetric analysis

**Figure 4.**
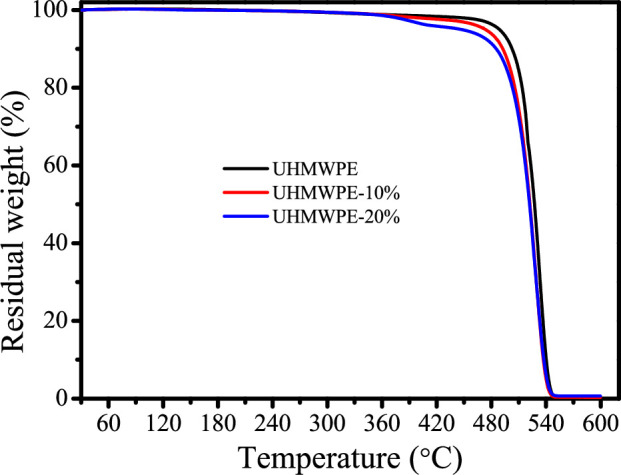
TGA curves of UHMWPE/PE-g-PHMG monofilaments.

The thermal degradation characteristics of UHMWPE/PE-g-PHMG monofilaments were examined through TGA. The relevant findings are illustrated in figure 4 and summarized in table 2. The temperature at which a 5 wt% mass loss is observed is referred to as the initial decomposition temperature (*T*_5%_). The temperature at which the monofilaments demonstrate the highest rate of thermal decomposition is designated as *T*_max_. It is significant to note that the mass of the blended monofilaments remains relatively stable up to 300℃, indicating thermal stability under the specified spinning conditions. For the UHMWPE monofilament, thermal degradation initiates at temperatures approaching 488.6℃, with a mass reduction of approximately 5%. This mass loss is probably due to the evaporation or degradation of low molecular weight compounds present within the monofilaments. As the temperature increases further, the degradation rate escalates markedly, reaching a peak at 528.6℃. In contrast to the UHMWPE monofilament, the UHMWPE-10% and UHMWPE-20% monofilaments exhibit lower *T*_5%_ values of 473.3 and 446.6℃, respectively, as well as lower *T*_max_ values of 525.1 and 522.4℃, respectively. These results indicate that the incorporation of PE-g-PHMG negatively impacts the thermal stability of the monofilaments.

**Table 2.** TGA data of UHMWPE/PE-g-PHMG monofilaments.

samples	*T_5%_*	*T*_max_
UHMWPE	488.6	528.6
UHMWPE-10%	473.3	525.1
UHMWPE-20%	446.6	522.4

### 3.5. Crystallization study

**Figure 5.**
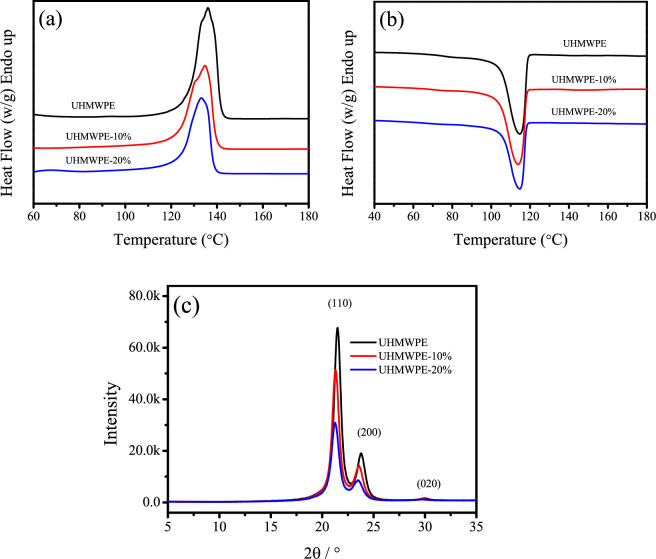
DSC heating curves (a), cooling curves (b) and XRD profiles (c) of UHMWPE/PE-g-PHMG monofilaments.

The impact of PE-g-PHMG content on the melting behaviour, crystallinity and crystalline structure of blend monofilaments was investigated using DSC and XRD techniques. The DSC heating and cooling curves are illustrated in figure 5a,b. Distinct endothermic peaks corresponding to the melting temperatures can be observed for blend monofilaments. XRD profiles are depicted in figure 5c, three prominent diffraction peaks: (110) at 2*θ* = 21.5° (200) at 2*θ* = 23.7° and (020) at 2*θ* = 29.9° are observed, corresponding to the orthorhombic phase of PE [31]. The XRD peak intensities decrease and the widths of the peaks increase with increasing PE-g-PHMG content, indicating the reduction of crystallinity and the formation of imperfect crystals.

The melting enthalpy (Δ*H*_m_), crystallinity (*X*_c_), crystallization enthalpy (Δ*H*_c_), average lattice spacing (*d*_hkl_) and average crystallite size (*D*_hkl_) of the blend monofilaments are presented in table 3. The crystallinity was estimated based on the ratio of the measured heat of fusion to the heat of fusion (293 J g^−1^) of 100% crystalline PE [25]. The values of crystallinity values obtained are 67.8, 58.7 and 49.5% for UHMWPE, UHMWPE-10% and UHMWPE-20%, respectively, as indicated in table 3. These results demonstrate that the presence of PE-g-PHMG components contributes to the kinetic hindrance of UHMWPE crystallization during the melt-spinning and hot-drawing processes. A similar trend is observed during the cooling process, where the crystallization enthalpy (Δ*H*_c_) decreases significantly with increasing PE-g-PHMG content, suggesting that PE-g-PHMG inhibits the crystallization of UHMWPE. The 2*θ* values corresponding to the (110) and (200) crystal planes in table 3 indicate a leftward shift of diffraction patterns with the addition of PE-g-PHMG, indicating that *d*_110_ and *d*_200_ increase with increasing PE-g-PHMG content. Furthermore, the average crystallite sizes, *D*_110_ and *D*_200_, decrease, suggesting that the presence of PE-g-PHMG inhibits the growth of crystals in the normal direction of the (110) and (200) crystal planes. From the above research results, it can be seen that the incorporation of PE-g-PHMG adversely affects the crystallinity and crystalline structure in the blend monofilaments.

**Table 3.** Summary of the melting, crystallization behaviour and structural parameters of crystal of blend monofilaments.

samples	heating		cooling	diffraction peak (hkl)	2*θ*	*d*_hkl_ (nm）	*D*_hkl_ (nm）
	Δ*H*_m_ (J g^−1^)	*X*_c_ (%)	Δ*H*_c_ (J g^−1^)				
UHMWPE	198.7	67.8	−217.7	110	21.489	0.413	11.1
				200	23.822	0.373	9.1
UHMWPE-10%	172.1	58.7	−167.9	110	21.299	0.417	10.7
				200	23.608	0.376	8.9
UHMWPE-20%	145.1	49.5	−140.8	110	21.260	0.417	9.8
				200	23.525	0.378	8.1

### 3.6. Effect of polyethylene-grafted poly(hexamethylene guanidine) on viscoelastic behaviour

**Figure 6.**
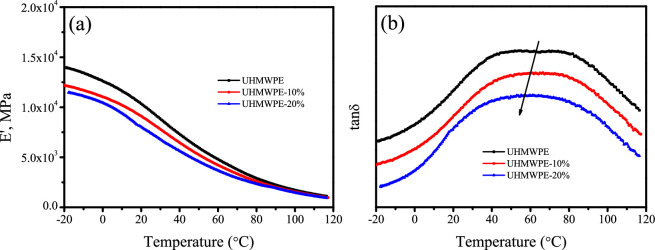
The variation of storage modulus (a) and tan δ (b) with temperature of UHMWPE/PE-g-PHMG blend monofilaments.

The viscoelastic behaviour of polymers is significantly affected by temperature. Dynamic mechanical analysis (DMA) is an effective technique for evaluating the dynamic viscoelastic response of UHMWPE/PE-g-PHMG blend monofilaments across a range of temperatures. The loss factor, represented as tan δ, is defined as the ratio of loss modulus (*E*″) to storage modulus (*E′*). Figure 6a depicts the variation of storage modulus (*E*′) with temperature of UHMWPE/PE-g-PHMG monofilaments. The *E*′ of all samples decreases with increasing temperature. This trend reflects the material’s ability to dissipate deformation energy and serves as an indicator of material rigidity. As temperature rises, the polymer chains undergo significant relaxation, resulting in increased mobility and a notable reduction in the storage modulus. Moreover, an increase in the content of PE-g-PHMG leads to a higher loss factor, as shown in figure 6a. The incorporation of PE-g-PHMG into the UHMWPE matrix disrupts crystallinity, which contributes to a decrease in the storage modulus. Consequently, the reduction in the storage modulus is associated with a decrease in the overall rigidity of the blend. Figure 6b depicts the variation of tan δ with temperature of UHMWPE/PE-g-PHMG monofilaments, revealing a broad loss peak in the range of 40−80℃, corresponding to the α relaxation of PE [32]. It has been reported that the α relaxation is related to the crystalline phase and due to the motion of the chains within the crystalline lamellae [33,34]. It can be seen that the position of loss peak shifts slightly towards lower temperatures with increasing PE-g-PHMG content, probably due to smaller constraints from lower crystallinity.

### 3.7. Effect of polyethylene-grafted poly(hexamethylene guanidine) on tensile behaviour

**Figure 7.**
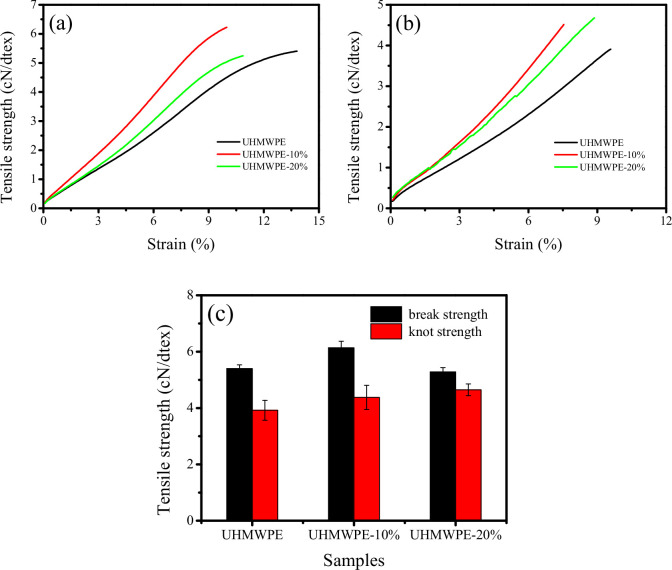
Tensile curves of both non-knotted (a), and knotted (b) blend monofilaments with a draw ratio of 8 and summary of breaking strength and knot strength (c).

Tensile curves for both non-knotted and knotted blend monofilaments with a draw ratio of 8 are illustrated in figure 7a,b. Furthermore, a summary of the breaking strength and knot strength is presented in figure 7c. The breaking strength and knot strength of the UHMWPE monofilament were 5.4 and 3.9 cN dtex⁻¹, respectively. For the UHMWPE-10% monofilament, these values increased by 13.0% (6.1 cN dtex⁻¹) and 12.8% (4.4 cN dtex⁻¹), respectively. However, a slight decrease in breaking strength (5.3 cN dtex⁻¹) and a continuous increase in knot strength (4.6 cN dtex⁻¹) were observed for the UHMWPE-20% monofilament. Notably, the UHMWPE-10% monofilament demonstrates a peak in breaking strength. It is essential to acknowledge that breaking strain and Young’s modulus are greatly influenced by monofilament diameter, so we do not consider the change in breaking strain and Young’s modulus. As can be seen from our previous results, an increase in PE-g-PHMG content decreases the crystallinity and rigidity, which in turn increases the tensile toughness of blend monofilaments. And the stress concentration area near the knot could undergo effective plastic deformation, which could improve the knot strength. Thus, the knot strengths increased with an increase in PE-g-PHMG content.

### 3.8. Antimicrobial properties of ultra-high molecular weight polyethylene/polyethylene-grafted poly(hexamethylene guanidine) monofilaments

**Figure 8.**
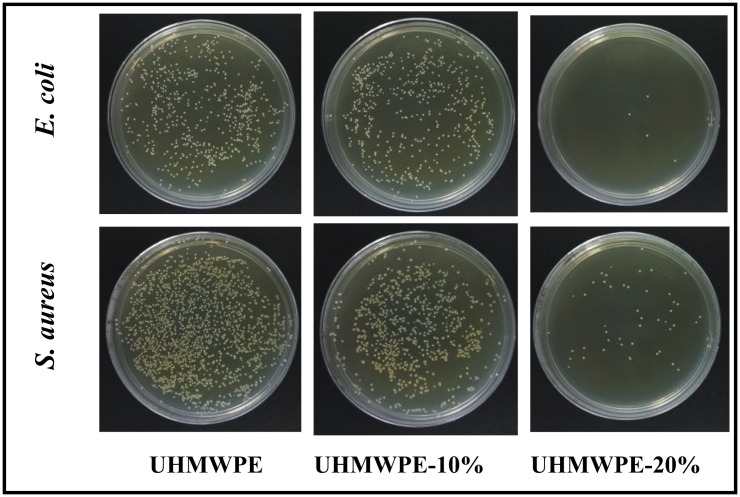
Antimicrobial activity of UHMWPE, UHMWPE-10% and UHMWPE-20% monofilaments against *E. coli* and *S. aureus*.

**Figure 9.**
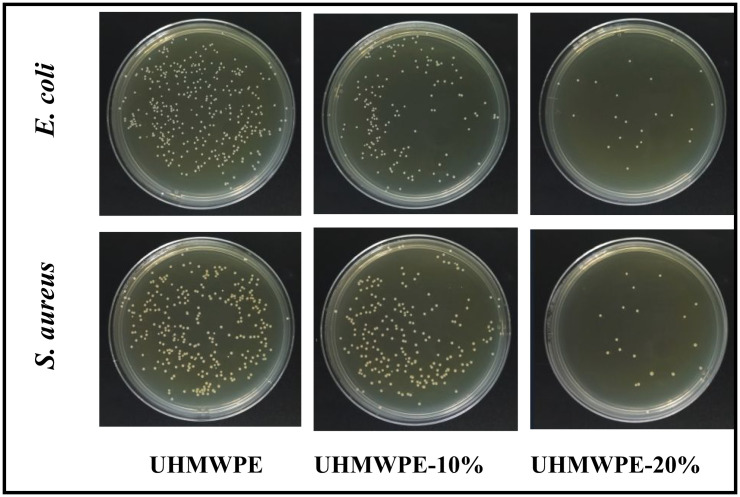
Antimicrobial activity of UHMWPE, UHMWPE-10% and UHMWPE-20% monofilaments after washing 30 times against *E. coli* and *S. aureus*.

The antimicrobial properties of blended monofilaments were evaluated using the shaking flask method. Figure 8 depicts the antimicrobial activity of UHMWPE, UHMWPE-10% and UHMWPE-20% monofilaments against *E. coli* and *S. aureus*. As can be seen, blend monofilaments showed significant differences in their antimicrobial effect. *Escherichia coli* colonies and *S. aureus* colonies were abundantly present on the UHMWPE plate, suggesting that UHMWPE alone exhibits limited antimicrobial activity. In contrast, the incorporation of PE-g-PHMG was associated with a reduction in the number of *E. coli* and *S. aureus* colonies, indicating that PE-g-PHMG possesses significant antimicrobial properties. An enhancement in antibacterial activity was observed with increasing concentrations of PE-g-PHMG. The calculated antimicrobial efficacy of UHMWPE-10% monofilaments against *E. coli* and *S. aureu*s was found to be 12.7 and 56.5%, respectively. While UHMWPE-20% monofilaments exhibited efficacy rates of 99.4 and 97.2% against *E. coli* and *S. aureus*. Furthermore, FTIR spectroscopy revealed an increase in the content of the PHMG component, which correlated with enhanced antimicrobial activity. Besides, the calculated antimicrobial efficacy of UHMWPE-10% monofilaments after washing 30 times against *E. coli* and *S. aureus* was found to be 59.1 and 26.6%, respectively, as shown in figure 9. And UHMWPE-20% monofilaments after washing 30 times exhibited efficacy rates of 92.7 and 92.5% against *E. coli* and *S. aureus*. When the total positive charge of PHMG component matches the negative charges of bacteria, the membrane of bacterial cells is destroyed, causing the leakage of intracellular contents and the death of bacteria.

## 4. Conclusion

In this study, PE-g-PHMG-modified UHMWPE monofilaments were synthesized, and their structural characteristics (including chemical and crystalline structures), as well as their thermal, mechanical and antibacterial properties were thoroughly examined. The presence of a characteristic absorption peak corresponding to the C=N groups of PHMG in the blend monofilaments confirmed the successful grafting of PHMG onto PE and its retention in the blend monofilaments following high-temperature melt spinning. The incorporation of PE-g-PHMG resulted in a decrease in the degree of crystallinity, average lattice spacing and crystallite size in the blend monofilaments, leading to relatively low rigidity and high toughness. Due to high toughness with increasing PE-g-PHMG content, the stress concentration area near the knot undergoes effective plastic deformation, which supports the transmission of force within a large strain, thereby improving the knot strength of the blend monofilaments. Furthermore, a gradual increase in antibacterial efficacy was observed with increasing PE-g-PHMG content, with inhibition rates of blend monofilaments against *E. coli* and *S. aureus* recorded at 99.4 and 98.2%, respectively. The successful development of monofilaments that exhibit both high knot strength and favourable antibacterial properties indicates their potential for widespread application in fisheries and aquaculture.

## Data Availability

The supporting information has been uploaded as the supplementary material [35]. All data can be accessed through the Dryad database [36].
